# A spectroscopy approach to the study of virus infection in the endophytic fungus *Epichloë festucae*

**DOI:** 10.1186/1743-422X-8-286

**Published:** 2011-06-08

**Authors:** Cristina Petisco, Balbino Garcia-Criado, Iñigo Zabalgogeazcoa, Beatriz R Vázquez-de-Aldana, Antonia Garcia-Ciudad

**Affiliations:** 1 Instituto de Recursos Naturales y Agrobiología de Salamanca, IRNASA-CSIC, Apdo. 257, 37071 Salamanca, Spain

**Keywords:** Near-infrared spectroscopy, viruses, fungal endophytes, *Epichloë festucae*, *Festuca rubra*

## Abstract

**Background:**

In this work we propose a rapid method based on visible and near-infrared (Vis-NIR) spectroscopy to determine the occurrence of double-stranded RNA (dsRNA) viruses in *Epichloë festucae *strains isolated from *Festuca rubra *plants. In addition, we examined the incidence of infections by *E. festucae *in populations of *F. rubra *collected in natural grasslands of Western Spain.

**Methods:**

Vis-NIR spectra (400-2498 nm) from 124 virus-infected and virus-free *E. festucae *isolates were recorded directly from ground and freeze-dried mycelium. To estimate how well the spectra for uninfected and infected fungal samples could be differentiated, we used partial least-squares discriminant analysis (PLS1-DA) and several data pre-treatments to develop calibration models.

**Results:**

Applying the best regression model, obtained with two sampling years and using standard normal variate (SNV) combined with first derivative transformation to a new validating data set (42 samples), we obtained a correct classification for 75% of the uninfected isolates and up to 86% of the infected isolates.

**Conclusions:**

The results obtained suggest that Vis-NIR spectroscopy is a promising technology for detection of viral infections in fungal samples when an alternative faster approach is desirable. It provides a tool adequately exact and more time- and cost-saving than the conventional reference analysis.

## Background

The perennial grass *Festuca rubra *is very common in semiarid grasslands of Western Spain, and wild populations of this species are often infected by the endophytic fungus *Epichloë festucae *[1,2]. In populations of *F. rubra *infected by *E. festucae*, most plants are asymptomatic and produce infected seeds, although a few plants may develop fungal stromata which block the emergence of flowering stems [3]. *Epichloë festucae *is considered a mutualistic fungus; *F. rubra *plants infected by *E. festucae *are more resistant to several species of insect herbivores, because they contain several types of alkaloids produced by the fungus, and have a better appearance and survival rates under stressful conditions than uninfected plants [3]. As a consequence the improvement of *F. rubra *cultivars with *Epichloë *endophytes is an objective of some turfgrass breeding programs [4].

Whereas viruses of plants have long been recognized as important components of plant biosystems, viruses of fungi have been largely ignored. The associations between fungal viruses and their hosts are similar to plant-endophyte associations, because many known fungal viruses cause no obvious symptoms [5,6]. Only a few fungal viruses are known to affect their hosts, one example is La France isometric virus (LIV), the causal agent of one of the most devastating diseases in the commercial production of the mushroom *Agaricus bisporus *[7].

The presence of two viruses with genomes of 5.2 kbp (EfV1) and 3.2 kbp (EfV2) has been described in *E. festucae*. EfV1 is a member of the genus *Victorivirus *(Fam. *Totiviridae*), and EfV2 is thought to be a member of the *Narnaviridae *family. Both viruses are efficiently transmitted to asexual spores produced by infected isolates of the fungal endophyte. However, no obvious phenotype is observed in virus infected isolates of *E. festucae *[8,9].

Although several methods, including enzyme-linked immunosorbent assay (ELISA), polymerase chain reaction (PCR), immunofluorescent assay and Western blotting have been used for the diagnosis of viral infections, none of them is ideal in terms of cost-effectiveness, speed, and accuracy. Currently, the rate of outbreak of emerging viruses is increasing and therefore the development and establishment of analytical methods for such viral infections are becoming more important [10].

Near-infrared (NIR) spectroscopy is a fast, accurate and non-destructive technique that does not require chemical reagents. Visible and NIR spectroscopy is the method which covers the region from 400 to 2500 nm. The absorption of molecules in the NIR region is due to combinations and overtones of vibration such as stretching and bending of hydrogen-bearing functional groups like -CH, -OH, and -NH [11]. The development of a calibration depends on a multivariate mathematical process based on a set of reference data which have been obtained by a standard chemical method. The process of calibration and its subsequent validation are an important part of NIR analysis [10].

Nowadays, NIR spectroscopy has been applied successfully in many fields such as agriculture, environment, and medicine, as well as in the pharmaceutical, chemical, petrochemical and food industries [12]. However, until recently NIR spectroscopy had not been used in virology. Some authors have reported attempts to use NIR spectroscopy for viral disease diagnoses, i.e., human immunodeficiency virus (HIV) [13,14], diagnosis of the presence of tobacco mosaic virus (TMV) in tomatoes [15], or virus infections in soybean [16]. The future should see an increasing use of NIR spectroscopy in virology for diagnosis, characterization of viruses, examination of the pathology of virus-associated diseases, measurement of virus load, and so on [10]. The detection and identification of viral infections by spectroscopic techniques promises to be of a great value because of their sensitivity, rapidity and low expenses [17].

The objective of this study was to develop models using spectral Vis-NIR reflectance measurements to discriminate between virus infected and virus free strains of the endophytic fungus *E. festucae*. This technique could be useful as a quick identification tool to give solid clues before confirmation by a genotypic method. In addition, a field study was done to determine the frequency of infection by the endophyte *E. festucae *in *F. rubra *plants, and the incidence of viruses in these populations of *E. festucae*.

## Methods

### Plant and fungal isolates

Isolates of *E. festucae *were obtained during the spring of 2008 from 161 plants of *F. rubra *collected at six different locations in natural semiarid grasslands of the province of Salamanca, in Western Spain. At each location between 21 and 34 plants were dug out, leaving a distance of at least 10 m from each other, and transported to the laboratory. *Epichloë festucae *was isolated from plant stems and leaf sheaths surface disinfected with a 15 minute treatment with a solution of 20% commercial bleach (1% active chlorine). After rinsing in sterile water, about 15 stem pieces were placed on each of 2 Petri plates containing potato dextrose agar (PDA) with 200 mg/l of chloramphenicol and incubated at 24°C in the dark. It takes about one week for *E. festucae *mycelium to emerge from plant fragments [18]; therefore, mycelia emerging from plant parts during the first 5 days after plating were discarded, since these were possibly other fungal species. A total of ninety-three isolates were identified as *E. festucae *on the basis of morphological characteristics of the cultures [19].

To produce enough mycelium for dsRNA extraction and NIR analyses, each isolate was grown on top of sterile cellophane disks layered on top of PDA plates. The mycelium obtained from eight plates was freeze-dried and ground in an electric coffee grinder.

In addition, for the NIR study 31 *E. festucae *isolates obtained from two locations (Servandez and Palancar) in the year 2006 were used. These isolates were also grown on top of sterile cellophane disks in eight PDA plates, and their mycelium was freeze-dried, but the grinding was done with liquid nitrogen in a mortar. These samples were included in the NIR analysis to add more universality, and to account for more variability (year, locations, pulverizing procedure of the samples, and age of the cultures: 30 days for 2006 samples and 40 days for 2008 samples), but were not considered for the estimation of endophytic and virus infection frequencies.

### Virus detection in *Epichloë *cultures

The presence of the viruses EfV1 and EfV2 was diagnosed using 93 isolates of *E. festucae *from 2008, and 31 from 2006 by means of the detection of the 5.1 and 3.2 kbp dsRNA molecules which correspond to the genomes or replicative intermediates of the viruses. The dsRNA was purified from 0.5 - 1 g of mycelium by CF-11 cellulose chromatography [20]. The products of each dsRNA extraction procedure were resolved by electrophoresis in 1% agarose gels. To avoid false negative diagnostics, all isolates in which no dsRNA was detected were analysed twice.

### Spectra acquisition

For the acquisition of spectra, five freeze-dried and ground cultures of each isolate were scanned on a NIRSystem 6500 scanning monochromator (FOSS NIRSystems, Silver Spring, MD) fitted with a sample transport module. The pulverized mycelium was placed on a circular sample cell with a quartz window (38 mm in diameter and 10 mm in thickness). ISIscan version 3.10 and WinISI 4 software (Infrasoft International, State College PA) were used to control and diagnose instrument performance and to manage the recorded spectra. Spectra from 400 to 2500 nm were acquired at 2 nm wavelength increments, obtaining 1050 data points for each spectrum. Visible and near infrared measurements were made in reflectance mode (R). The average of the five spectra obtained from each isolate was used in the following analyses. A total of 124 mean spectra were recorded as log (1/R), where R is the intensity of reflected light at each wavelength. The laboratory temperature was kept at 24°C during NIR measurements.

### Data analysis

For data analysis, spectral (log 1/R) files were exported from WinISI in NSAS format to The Unscrambler software (v. 9.6; CAMO Software AS, Oslo, Norway). Calibrations generated for the classification of virus infected and virus free isolates were developed and evaluated with separated calibration and prediction sample sets. The calibration set consisted of two-thirds of the samples (n = 82 samples; 62 samples of 2008 and 20 of 2006), and the validation set consisted of the remaining third (n = 42 samples; 31 samples of 2008 and 11 of 2006). Samples were assigned to each set based on their position in the spectral file; thus, every third sample was used for external validation and the remainder for calibration.

Previous to the development of the classification models, principal component analysis (PCA) was made using The Unscrambler software to inspect spectral patterns for improved understanding, and to detect outliers or any clustering of the data [21]. A supervised pattern recognition technique, with *a priori *knowledge about the category membership of samples, was used in the qualitative analysis. Partial least-squares discriminant analysis (PLS-DA) was the method used to generate calibrations for the classification. It is a method that compresses the spectral data into orthogonal structures called factors, which describes the maximum covariance between the spectral information and the reference values. Successful compression using only a few factors is often possible in spectroscopy because the numerous data points are highly co-linear [21,22].

Because categorical predictor variables cannot be entered directly into a regression model and be meaningfully interpreted, some other method of dealing with information of this type must be developed. In this paper, PLS1-DA onto a dummy variable [23] was used for discrimination. In this method, a calibration matrix is developed using dummy variables assigning an arbitrary number if the sample belong to a particular group or if it does not. A dummy value of 1 was assigned for infected samples and 2 for uninfected samples. The PLS1 models were developed by regression of the spectral data (raw or transformed) against the assigned reference values (dummy variables), and then tested for accuracy by using it to predict samples belonging to the validation set. The optimal number of factors in the regression models was determined by full cross-validation. A sample was classified as infected if its predicted value was between 0.5 and 1.5, and as non-infected if the predicted value was between 1.5 and 2.5. The accuracy of classification models was assessed on the basis of the percentages of correct classification.

Pre-treatments of the spectral data are often applied in an effort to improve calibration accuracy. In order to obtain the best discrimination model, nine different types of spectral pre-treatments were tested. These included no treatment (raw spectral data), multiplicative scatter correction (MSC), standard normal variate (SNV), first derivative (1D), second derivative (2D), and MSC or SNV followed by derivative transformations (Savistsky Golay method). Particular derivative parameters used were 1D (5,5,1) and 2D (6,6,2), in which the first and second numbers are the left and right number of side points, and the third is the polynomial order. A more detailed description of these data pre-treatments can be found in Petisco et al. [24].

## Results

### Endophytic infection in populations of *F. rubra *and incidence of viruses in *E. festucae*

In the six populations analyzed in 2008 we found that an average of 59% of the plants was infected by *E. festucae*. The infection frequencies ranged from 24% to 87% (Table 1).

**Table 1:** Incidence of infections of *E.festucae *in *F. rubra *and of mycoviruses in *E. festucae *isolates

Location	Endophytic infection		Virus infection	
	
	N° of plants	% plants infected	N° of isolates	% isolates infected
Berrocal	34	59	20	50
Los Valles	21	81	17	82
Servández	25	44	11	64
Llen	24	87	21	19
Membribe	28	61	17	53
Valeros	29	24	7	71
				
Mean	27	59	15	57

The samples considered for the study of the incidence of infections correspond to the year 2008

**Figure 1:**
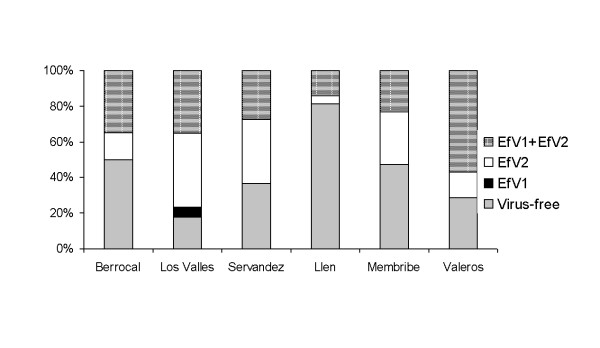
**Frequency of EfV1 and EfV2 virus infections (%) in *E. festucae *populations**.

The incidence of virus infections was relatively high among *E. festucae *isolates, 57% of those obtained in 2008 contained dsRNA molecules indicating the presence of EfV1 and/or EfV2. The incidence of virus infection ranged from 19% in Llen to 82% in Los Valles (Table 1). With the exception of Llen, all populations had incidences greater than 50%. EfV2 and coinfections by EfV1 and EfV2 were common in isolates from most locations (Figure 1). Only one isolate, found in Los Valles, was infected only by the virus EfV1.

### Spectra characterisation

**Figure 2:**
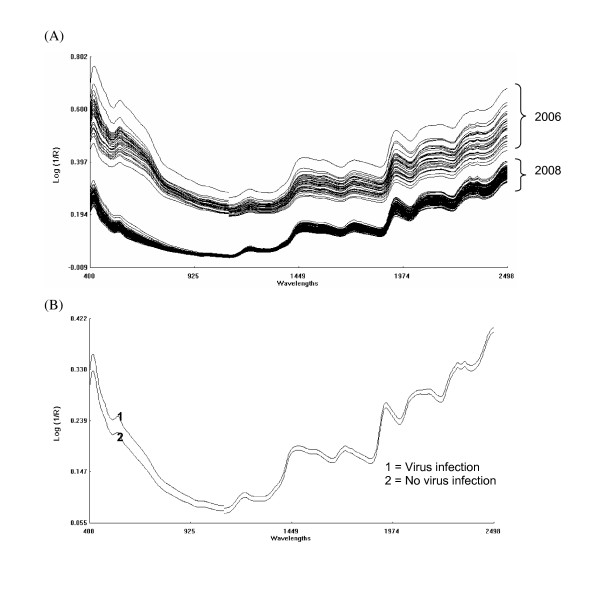
**Raw spectra**. (A) all isolates from 2006 and 2008 years; (B) average spectra of virus infected and uninfected isolates of *E. festucae*

The registered Vis-NIR spectra of the 2008 isolates had a decreased absorption compared to the spectra of the 2006 isolates (Figure 2A), and the spectra of virus free isolates displayed a decreased absorption relative to the virus infected isolates (Figure 2B).

Multivariate statistical techniques were needed for the analysis of Vis-NIR data because of the little difference between the spectra.

### PCA analysis

**Figure 3:**
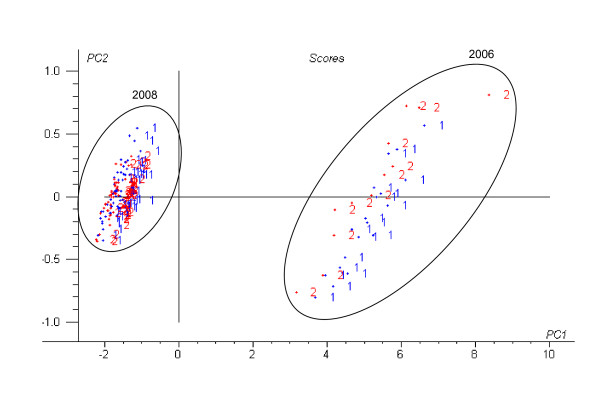
**Scores plot of the first two PCs of all *E. festucae *samples**. (log 1/R Vis-NIR spectra; 1 = infected; 2 = uninfected)

Figure 3 shows the PCA scores (PC1 vs PC2) for *E. festucae *samples in the vis-NIR region using the raw spectra. Since no unusual or outlying samples were detected, all samples were used in subsequent chemometric analyses. The first two PCs explained 99.8% of the total variation in the raw spectra. As it can be noticed from the scores biplot for components 1 and 2, there were two clearly separated groups, corresponding to the isolates collected in 2006 or in 2008 (Figure 3). However, the infected and uninfected classes remained considerably overlapped in the 2008 samples.

### Discrimination based on PLS1-DA

**Figure 4:**
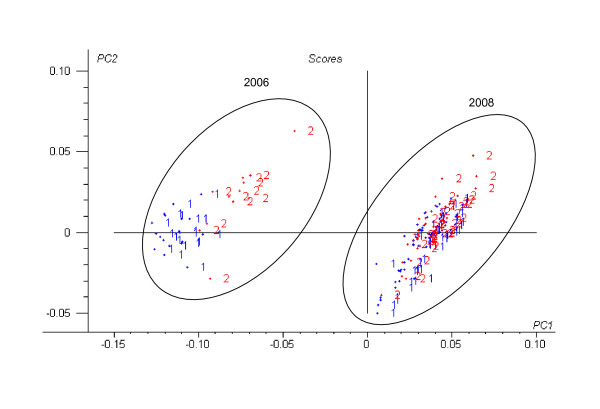
**PLS scores plot of the first two PCs of all *E. festucae *samples**. (SNV+1D Vis-NIR spectra; 1 = infected; 2 = uninfected)

The PLS1 classification rates (percent of correct classification) according to infection for the external validation sets are shown in Table 2. The models were developed using the Vis-NIR raw data and after pre-processing the spectra with a combination of the multiplicative scatter correction (MSC), standard normal variate (SNV), first derivative (1D) and second derivative (2D). The effect of these mathematical treatments on the prediction quality of the models is shown in Table 2. The scores plot (Figure 4) of the first two PLS loadings of the PLS1 model involving the use of standard normal variate and first derivative (SNV+1D) pre-treatments was similar to the PCA score plot (Figure 3); however, separation of samples according to infection was more obvious, particularly in the samples from 2006.

**Table 2:** PLS1 method to estimate the correct classification (%) of virus infection in *E.festucae *isolates

Data pre-treatment	2006 (*n *= 11)			2008 (*n *= 31)			2006 + 2008 (*n *= 42)		
	
	F^a^	Virus (*n *= 6)	No virus (*n *= 5)	F^a^	Virus (*n *= 16)	No virus (*n *= 15)	F^a^	Virus (*n *= 22)	No virus (*n *= 20)
Raw	4	100	80	1	75	47	6	73	65
MSC^b^	3	100	80	4	81	60	5	77	70
SNV^c^	3	100	80	4	81	60	6	82	75
1D^d^	2	100	80	1	62	47	5	73	75
MSC^b^+1D^d^	2	100	80	2	**81**	**67**	4	73	75
SNV^c^+1D^d^	2	100	80	2	**81**	**67**	3	**86**	**75**
2D^e^	4	**100**	**100**	1	62	53	4	73	65
MSC^b^+2D^e^	1	**100**	**100**	3	75	60	4	73	55
SNV^c^+2D^e^	1	**100**	**100**	3	75	60	4	82	75

Results of the external validation for individual and combined years (2006 and 2008) using different data pre-treatments. Bold type indicates the best classifications obtained for detection of virus infection

^a ^Number of PLS factors

^b ^Multiplicative scatter correction

^c ^Standard normal variate

^d ^First derivative

^e ^Second derivative

The best results were achieved in samples from 2006; in this case, using one PLS factor, we obtained a percentage of correct classification of 100% in both classes of *E. festucae *isolates (infected and uninfected) when applied the data transformed to 2D or its combination with MSC or SNV (Table 2). Respect to the results obtained with the 2008 samples, using two PLS factors, 13 out of 16 samples were correctly assigned to the infected class (81%) whereas 10 out of 15 correct assignments were made in the uninfected class (67%). These results were obtained after transforming the raw spectra to MSC+1D and SNV+1D pre-treatments. For the model including both samples of 2006 and 2008, the best pre-processing was SNV+1D. In this case 86% of the infected samples and 75% of the uninfected samples were correctly predicted using three PLS factors.

## Discussion

The 59% incidence of endophytic infection found in this work is slightly lower than that reported in other surveys in similar ecosystems [1], but confirms that the association between *F. rubra *and the endophyte *E. festucae *is a common event in grasslands of Western Spain (Table 1). *Epichloë festucae *is very efficiently transmitted to the seeds of infected plants, and no other mechanism of transmission is known for this fungus to occur in natural populations [3]. Infected plants originate from the seeds of infected parents, or from the vegetative expansion of infected ramets [2]. Because of this, the high infection rate observed suggests that in this environment the fungal infection might be either neutral or beneficial for the plants, otherwise infected plants would not be the majority in these natural populations. Although grass-endophyte associations have been associated with livestock toxicosis [3], the contents of the ergovaline alkaloid are below toxic levels in these semiarid ecosystems [25]. In addition, although the endophytic infection rate is high in *F. rubra*, these pastures are rich in other plant species and as a result, the ingestion of toxic plants is diluted with other plant species.

The high frequency of virus infection (Table 1 Figure 1) may be explained by the efficient transmission of both viruses during the asexual reproduction of *E. festucae*, which is the predominant mode of reproduction of the fungus [9]. The effect of the *Epichloë *viruses upon their host fungus and plant host is yet unknown.

The raw spectra show almost identical patterns for all isolate samples with strong absorption bands around 1440 nm (O-H first overtone) and 1950 nm (O-H combination bands) (Figure 2). Other absorption bands at 1200, 1720, 2100, 2310 and 2352 nm are related to the C-H bond vibration, and arise from oil, protein and starch or cellulose components [26].

Prior to discriminant analysis, principal component analysis (PCA) was used to obtain preliminary indications of the possible clustering of the samples in the two classes: fungal isolates infected and uninfected by viruses. We found two sample clusters in the PCA and PLS1 scores plots (Figures 3 and 4) corresponding to the 2006 and 2008 samples which could be related to the different way to processing both sample sets. Although the inclusion of heterogeneity in the samples may reduce the predictive model accuracy it provides variability and therefore universality to the developed NIR models. Models based on raw spectra were slightly worse than those based on transformed spectra with MSC or SNV combined with derivatives. The raw data, or the data transformed only with MSC or SNV, increased the number of optimum factors, and reduced the percentages of correct classification in the validation sets (Table 2). The use of these data pre-treatments was useful for removing physical spectral information (due to particle size), so that PLS1 models were performed based mainly on chemical spectral information.

The differences in classification between years could be due to the different ways used for grinding the fungal samples each year, since the particle size affects strongly to the absorbance of the spectra. Also, culture age was different for the 2006 and 2008 samples. Therefore, for best results it seems very important to process all the samples exactly in the same way, using cultures of the same age and pulverizing the freeze-dried mycelium in liquid nitrogen. In addition, the better separation of virus infected and virus free samples in 2006 could be due to the fact that among the 93 samples of 2008, infections by EfV2, as well as mixed infections by both viruses occurred. In contrast, all the virus infected samples from 2006 were infected by the two viruses, being their virus load higher. Therefore the best results of 2006 could also be related to a lower variability in samples.

*Epichloë *virus genomes are composed of dsRNA, and high molecular weight dsRNA is an specific virus-related compound which is absent from virus-free plants and fungi [20]. The dsRNA concentration of virus infected *E. festucae *strains is high, particularly in those infected by EfV1 [9]. Therefore, it is possible that the dsRNA present in virus infected cells is the reason why the NIR spectra of virus infected and virus free strains of *Epichloë *can be discriminated.

Experience with NIR data has shown that PLS-DA can give good prediction results in the classification of similar fungal species [24,27] or seeds damaged by fungi [28]. However, NIR spectroscopy has hardly been used in virology and its value for diagnostics is beginning to be realized [10]. This technique using the PLS-DA method has been used for the diagnosis of HIV-1 infection; in this case the results obtained by the NIR spectroscopic model (600-1000 nm) yielded a good correlation with those obtained with the reference method (HIV-1 p24 ELISA) [13]. Xu et al. [15] used multispectral reflectance measurements to make an early discrimination between healthy and TMV infected tomato plants achieving good results. Other methods, such as soft independent modelling of class analogy (SIMCA), have also been used in the detection of viruses. Bahamani et al. [14] showed a reliable separation of HIV-1-infected plasma from healthy plasma, and Jinendra et al. [16] obtained a calibration model which predicted the infection by soybean mosaic virus (SMV) in soybean plant samples with 91.6% sensitivity and 95.8% specificity when the second order derivative of individual plant averaged spectra were used. We do not show the results of the SIMCA models because they were not as good for the detection of virus infection as those developed by PLS-DA. This agrees with the worse results obtained with SIMCA versus PLS-DA for the classification of fungal endophytes in a previous paper [24].

This study is the first to demonstrate that Vis-NIR spectroscopy combined with PLS1-DA method is able to detect viral infections in a fungus, *E. festucae*. This technique might be useful for other situations were fungi are infected by viruses, for example for the diagnosis of La France disease in mushrooms. Currently the detection of fungal viruses is based on techniques like CF11 chromatography, which are a laborious conventional method of analysis.

## Conclusions

The results of this work suggest relatively high levels of infection by *E. festucae *in *F. rubra *plants and in *E. festucae *isolates by viruses. The application of the PLS-DA algorithm to visible and near-infrared spectra of fungal isolates was found to be a promising method for the rapid detection of virus infection.. In all cases, pre-processing was advantageous to improve interpretability and classification ability.

Further work will be necessary to standardize sample presentation and spectral collection and to optimize the calibration procedures for validating the obtained calibration models with independent samples from other locations or years. Anyway, although the results obtained in this study could be considered as preliminary, the technique may be considered as promising for future studies including large number of samples infected with other viruses associated to plant pathogenic fungi.

## Competing interests

The authors declare that they have no competing interests.

## Authors' contributions

CP carried out the experiments and drafted the manuscript. AGC and IZ supervised all experiments and provided discussion for the preparation of the final report. IZ and BGC participated in sample collection and BRVA participated in the revision of the manuscript. All authors have read and approved the final manuscript.

## References

[B1] Zabalgogeazcoa I Vazquez de Aldana BR Garcia Criado B Garcia Ciudad A The infection of *Festuca rubra *by the fungal endophyte *Epichloë festucae *in Mediterranean permanent grasslands Grass Forage Sci 1999 54 91 95 10.1046/j.1365-2494.1999.00155.x

[B2] Arroyo R Martínez Zapater JM García Criado B Zabalgogeazcoa I The genetic structure of natural populations of the fungal endophyte *Epichloë festucae* Mol Ecol 2002 11 355 364 10.1046/j.0962-1083.2001.01456.x 11918775

[B3] Schardl CL Leuchtmann A Spiering MJ Symbioses of grasses with seedborne fungal endophytes Annu Rev Plant Biol 2004 55 315 340 10.1146/annurev.arplant.55.031903.141735 15377223

[B4] Brilman LA Roberts CA, West CP, Spiers DE Endophytes in turfgrass cultivars Neotyphodium in Cool Season Grasses 2005 USA: Blackwell Publishing 341 349

[B5] Ghabrial SA Suzuki N Mahy BMJ, Van Regenmortel MHV Fungal viruses Encyclopedia of virology 2008 2 3 Oxford: Elsevier 284 291

[B6] Pearson MN Beever RE Boine B Arthur K Mycoviruses of filamentous fungi and their relevance to plant pathology Mol Plant Pathol 2009 10 115 128 10.1111/j.1364-3703.2008.00503.x 19161358 PMC6640375

[B7] Romaine CP Goodin MM Tavantzis SM Unravelling the viral complex associated with La France disease of the cultivated mushroom, *Agaricus bisporus* dsRNA genetic elements. Concepts and applications in agriculture, forestry, and medicine 2002 Boca Raton: CRC Press 237 257

[B8] Zabalgogeazcoa I Benito EP Garcia Ciudad A Garcia Criado B Eslava AP Double-stranded RNA and virus-like particles in the grass endophyte *Epichloë festucae* Mycol Res 1998 102 914 918 10.1017/S0953756297005819

[B9] Romo M Leuchtmann A Garcia B Zabalgogeazcoa I A totivirus infecting the mutualistic fungal endophyte *Epichloë festucae* Virus Res 2007 124 38 43 10.1016/j.virusres.2006.09.008 17081641

[B10] Sakudo A Suganuma Y Kobayashi T Onodera T Ikuta K Near-infrared spectroscopy: Promising diagnostic tool for viral infections Biochem Bioph Res Co 2006 341 279 284 10.1016/j.bbrc.2005.12.153 PMC7092872 16414011

[B11] Osborne BG Fearn T Hindle PT Practical NIR spectroscopy with applications in food and beverage analysis 1993 UK: Longman Group

[B12] Siesler HW Ozaki Y Kawata S Heise HM Near-Infrared Spectroscopy, Principles, Instruments, Applications 2002 Weinheim: Wiley-VCH

[B13] Sakudo A Tsenkova R Onozuka T Morita K Li S Warachit J Iwabu Y Li G Onodera T Ikuta K A novel diagnostic method for human immunodeficiency virus type-1 in plasma by near-infrared spectroscopy Microbiol Immunol 2005 49 695 701 16034213 10.1111/j.1348-0421.2005.tb03648.x

[B14] Bahmani MK Khosravi A Miri R Iwabu Y Ikuta K Sakudo A Spectroscopic characterization of human immunodeficiency virus type-1-infected plasma by principal component analysis and soft independent modelling of class analogy of visible and near-infrared spectra Mol Med Rep 2009 2 805 809 10.3892/mmr_00000176 21475905

[B15] Xu H Zhu S Ying Y Jiang H the Society of Photo-Optical Instrumentation Engineers Application of multispectral reflectance for early detection of tomato disease Proceedings of the Society of Photo-Optical Instrumentation Engineers: 3-4 October 2006; Boston 2006 Bellingham: ETATS-UNIS 63810R.1 63810R.8

[B16] Jinendra B Tamaki K Kuroki S Vassileva M Yoshida S Tsenkova R Near infrared spectroscopy and aquaphotomics: Novel approach for rapid in vivo diagnosis of virus infected soybean Biochem Bioph Res Co 2010 397 685 690 10.1016/j.bbrc.2010.06.007 20570650

[B17] Erukhimovitch V Karpasasa M Huleihel M Spectroscopic detection and identification of infected cells with herpes viruses Biopolymers 2008 91 61 67 10.1002/bip.21082 18932269

[B18] Zabalgogeazcoa I Romo M Keck E Vázquez de Aldana BR García Ciudad A García Criado B The infection of *Festuca rubra *subsp. *pruinosa *by *Epichloë festucae* Grass Forage Sci 2006 61 71 76 10.1111/j.1365-2494.2006.00509.x

[B19] Leuchtmann A Schardl CL Siegel MR Sexual compatibility and taxonomy of a new species of *Epichloë *symbiotic with fine fescue grasses Mycologia 1994 86 802 812 10.2307/3760595

[B20] Morris TJ Dodds JA Hillman B Jordan RL Lommel SE Tamak SE Viral specific dsRNA: diagnostic value for plant virus disease identification Plant Mol Biol Rep 1983 1 27 30

[B21] Næs T Isaksson T Fearn T Davies T A user-friendly guide to multivariate calibration and classification 2002 Chichester: NIR Publications

[B22] Martens H Martens M Multivariate analysis of quality. An introduction 2001 Chichester: John Wiley and Sons Ltd

[B23] Esbensen KH Multivariate Data Analysis in Practice. An Introduction to Multivariate Data Analysis and Experimental Design 2002 Norway: CAMO Process AS

[B24] Petisco C Downey G Murray I Zabalgogeazcoa I Garcia-Criado B Garcia-Ciudad A Direct classification of related species of fungal endophytes (*Epichloë *spp.) using visible and near-infrared spectroscopy and multivariate analysis FEMS Microbiol Lett 2008 284 135 141 10.1111/j.1574-6968.2008.01186.x 18492058

[B25] Vazquez-de-Aldana BR Zabalgogeazcoa I Rubio de Casas R García-Ciudad A García-Criado B Relationships between the genetic distance of *Epichloë festucae *isolates and the ergovaline and peramine contents of their *Festuca rubra *hosts Ann Appl Biol 2010 156 51 61 10.1111/j.1744-7348.2009.00360.x

[B26] Shenk JS Workman J Westerhaus MO Burns DA, Ciurczak EW Application of NIR spectroscopy to agricultural products Handbook of NIR Analysis 1992 New York: Marcel Dekker Inc 383 431

[B27] Decker M Nielsen PV Martens H Near-infrared spectra of *Penicillium camemberti *strains separated by extended multiplicative signal correction improved prediction of physical and chemical variation Appl Spectrosc 2005 59 56 68 10.1366/0003702052940486 15720739

[B28] Wang D Dowell FE Ram MS Schapaugh WT Classification of fungal-damaged soybean seeds using near-infrared spectroscopy Int J Food Prop 2004 7 75 82 10.1081/JFP-120022981

